# Predictors of intracranial hemorrhage in patients with atrial fibrillation treated with oral anticoagulants: Insights from the GARFIELD‐AF and ORBIT‐AF registries

**DOI:** 10.1002/clc.24109

**Published:** 2023-08-18

**Authors:** Toon Wei Lim, Alan John Camm, Saverio Virdone, Daniel E. Singer, Jean P. Bassand, Gregg C. Fonarow, Keith A. A. Fox, Michael Ezekowitz, Bernard J. Gersh, Gloria Kayani, Elaine M. Hylek, Ajay K. Kakkar, Kenneth W. Mahaffey, Karen S. Pieper, Eric D. Peterson, Jonathan P. Piccini, for the GARFIELD‐AF and ORBIT‐AF Investigators

**Affiliations:** ^1^ National Heart Centre Singapore Singapore; ^2^ National University Hospital Singapore Singapore; ^3^ Cardiology Clinical Academic Group Molecular & Clinical Sciences Institute St. George's University of London London UK; ^4^ Thrombosis Research Institute London UK; ^5^ Massachusetts General Hospital and Harvard Medical School Boston Massachusetts USA; ^6^ Department of Cardiology University of Besançon Besançon France; ^7^ Ronald Reagan‐UCLA Medical Center Los Angeles California USA; ^8^ Department of Cardiovascular Science Centre for Cardiovascular Science, University of Edinburgh Edinburgh UK; ^9^ Sidney Kimmel Medical School Thomas Jefferson University Philadelphia Pennsylvania USA; ^10^ Mayo Clinic College of Medicine and Science Rochester Minnesota USA; ^11^ Department of Medicine Boston University School of Medicine Boston Massachusetts USA; ^12^ Department of Surgery University College London London UK; ^13^ Stanford Center for Clinical Research Stanford School of Medicine Stanford California USA; ^14^ Department of Cardiac Electrophysiology Duke Clinical Research Institute Durham North Carolina USA; ^15^ Duke University School of Medicine Durham North Carolina USA

**Keywords:** anticoagulation, atrial fibrillation (AF), chronic kidney disease, intracranial hemorrhage (ICH), nonvitamin K antagonist (NOAC), oral anticoagulant (OAC), real‐world evidence (RWE), risk prediction, vitamin K antagonist (VKA)

## Abstract

**Background:**

An unmet need exists to reliably predict the risk of intracranial hemorrhage (ICH) in patients with atrial fibrillation (AF) treated with oral anticoagulants (OACs).

**Hypothesis:**

An externally validated model improves ICH risk stratification.

**Methods:**

Independent factors associated with ICH were identified by Cox proportional hazard modeling, using pooled data from the GARFIELD‐AF (Global Anticoagulant Registry in the FIELD‐Atrial Fibrillation) and ORBIT‐AF (Outcomes Registry for Better Informed Treatment of Atrial Fibrillation) registries. A predictive model was developed and validated by bootstrap sampling and by independent data from the Danish National Patient Register.

**Results:**

In the combined training data set, 284 of 53 878 anticoagulated patients had ICH over a 2‐year period (0.31 per 100 person‐years; 95% confidence interval [CI]: 0.28–0.35). Independent predictors of ICH included: older age, prior stroke or transient ischemic attack, concomitant antiplatelet (AP) use, and moderate‐to‐severe chronic kidney disease (CKD). Vitamin K antagonists (VKAs) were associated with a significantly higher risk of ICH compared with non‐VKA oral anticoagulants (NOACs) (adjusted hazard ratio: 1.61; 95% CI: 1.25–2.08; *p* = .0002). The ability of the model to discriminate individuals in the training set with and without ICH was fair (optimism‐corrected *C*‐statistic: 0.68; 95% CI: 0.65–0.71) and outperformed three previously published methods. Calibration between predicted and observed ICH probabilities was good in both training and validation data sets.

**Conclusions:**

Age, prior ischemic events, concomitant AP therapy, and CKD were important risk factors for ICH in anticoagulated AF patients. Moreover, ICH was more frequent in patients receiving VKA compared to NOAC. The new validated model is a step toward mitigating this potentially lethal complication.

## Graphical abstract

Over a 2‐year follow‐up period, intracranial hemorrhage (ICH) occurred in 284 out of 53 261 anticoagulated patients with atrial fibrillation from GARFIELD‐AF (Global Anticoagulant Registry in the FIELD) and ORBIT‐AF (Outcomes Registry for Better Informed Treatment of Atrial Fibrillation II) registries. A new model for ICH prediction was developed based on age, prior stroke or transient ischemic attack, vitamin K antagonist (VKAs) versus non‐VKA oral anticoagulants therapy, concomitant antiplatelet use, moderate‐to‐severe chronic kidney disease, and vascular disease. Discrimination for ICH was better than widely used scores for major bleeding whose use for ICH prediction has been published. This model may therefore aid risk stratification and shared decision‐making among clinicians and patients. Three examples of the score for patients with different risk factors are shown.

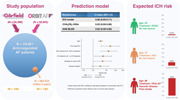

## 1 INTRODUCTION

Patients with atrial fibrillation (AF) have five times higher risk of stroke and two times higher risk of all‐cause mortality compared to the age‐matched general population.^1^ Standard of care for mitigating stroke risk is prophylactic oral anticoagulant (OAC) therapy using vitamin K antagonists (VKAs) or non‐VKA oral anticoagulants (NOACs). However, the benefit of these drugs must be balanced against an increased risk of bleeding. In particular, intracranial hemorrhage (ICH) is responsible for most of the deaths and disability linked to OAC‐associated bleeding.^2^ In previous meta‐analyses, rates of ICH‐related deaths were up to twice as high in AF patients using VKAs compared to those using NOACs, including when on concomitant aspirin therapy.^3^, ^4^, ^5^

Predictors of ICH ascertained in clinical trials of OAC include aspirin use, older age, and prior stroke/transient ischemic attack (TIA).^6^, ^7^ Clinical trials are often conducted in selected subjects. However, observational studies which can provide information about ICH risk factors that are relevant in everyday clinical settings^8^ have had limited power due to the rarity of the event.

The objectives of the present analysis were: (1) identifying factors that confer an increased risk of ICH in patients with AF, and on either VKA or NOAC therapy; (2) developing a predictive model using real‐world data from multiple countries.

## 2 METHODS

### 2.1 Registry designs and participants

Design and methodology of the GARFIELD‐AF (Global Anticoagulant Registry in the FIELD‐Atrial Fibrillation) and ORBIT‐AF (Outcomes Registry for Better Informed Treatment of Atrial Fibrillation) registries have been reported^9^, ^10^, ^11^ and are summarized in Table 1.

**Table 1:** Overview of GARFIELD‐AF and ORBIT‐AF I and II registries.

	GARFIELD‐AF	ORBIT‐AF I	ORBIT‐AF II
Enrollment period	2010–2016	2010–2011	2013–2016
Geography	Worldwide	USA	USA
No. of patients	52 000	10 000	13 400
Age	>18 years	>21 years	>21 years
AF criteria^a^	New onset	New onset or prevalent	New onset or new NOAC (<3 months)
AF diagnosis	Nonvalvular	Valvular or nonvalvular, ECG confirmed	Valvular or nonvalvular, ECG confirmed
Stroke risk	One additional risk factor	No requirement	No requirement
Exclusion criteria	Valvular or reversible AF	Reversible AF Life expectancy <6 months	Reversible AF Life expectancy <6 months

Abbreviations: AF, atrial fibrillation; ECG, electrocardiogram; GARFIELD‐AF, Global Anticoagulant Registry in the FIELD‐Atrial Fibrillation; NOAC, nonvitamin K antagonist; ORBIT‐AF, Outcomes Registry for Better Informed Treatment of Atrial Fibrillation.

^a^ New onset: Enrolled within 6 weeks of diagnosis.

Data from scheduled or unscheduled visits were recorded in the data capture system. When patients were unable to attend a regularly scheduled visit, attempts were made to contact the patient, collect relevant endpoint information, and confirm via medical records. Clinical data in both registries were collected at 6‐month intervals for at least 2 years after enrollment.

Independent ethics committee and hospital‐based institutional review board approvals were obtained for all patient data. The registries were conducted in accordance with the principles of the Declaration of Helsinki, and all participants provided written informed consent. The authors confirm that this manuscript follows STROBE (Strengthening the Reporting of Observational studies in Epidemiology) recommendations for reporting observational studies.

### 2.2 Statistical analyses

The primary outcome in the pooled registries was ICH over a 2‐year follow‐up. In GARFIELD‐AF, ICH was defined as primary ICH or major bleeding in one or more intracranial sites (intracerebral, intraventricular, subarachnoid, subdural, epidural, or site unknown). In ORBIT‐AF I and ORBIT‐AF II, ICH was defined as intracranial bleeding. Patients not treated with OAC or with unavailable follow‐up information were excluded from the analyses.

Baseline characteristics are presented for patients who did and did not develop ICH. Categorical variables are presented as frequency (%) and were compared by Pearson *χ^2^* or exact test as appropriate. Continuous variables were summarized as medians (25th, 75th percentile) and compared by Wilcoxon rank‐sum test. Prespecified potential predictors of ICH included a comprehensive set of demographic variables, smoking status, alcohol intake, clinical characteristics, and treatment factors (Supporting Information: Table S1). Only factors recorded in all three registries were considered. ICH event rates were estimated per 100 person‐years. Predictors of ICH were identified using least absolute shrinkage and selection operator (LASSO) methodology, including only the first occurrence of ICH in the model. A Cox proportional hazards model was fitted with the selected parameters, applying 30‐fold cross‐validation. All continuous covariates were tested for linearity, and appropriate transformations were applied when needed. Schoenfeld residuals, interaction of each selected covariate with time, and graphical methods were used to check the proportional hazard assumption.

The model for ICH prediction was developed according to the TRIPOD (Transparent Reporting of a multivariable prediction model for Individual Prognosis Or Diagnosis) checklist.^12^ Performance was evaluated internally by calculating optimism‐corrected *C*‐statistics. Because ICH is a rare event, it was not feasible to develop a split sample model. Instead, 100 bootstrap samples were constructed to obtain an optimism‐corrected *C*‐statistic to account for the overestimation bias of the model's performance in an external population.

For comparison, the predictive performances of CHA_2_DS_2_‐VASc (congestive heart failure, hypertension, age ≥75 [doubled], diabetes, stroke [doubled], vascular disease, age 65–74 and sex category [female]), HAS‐BLED (Hypertension, Abnormal Renal/Liver Function, Stroke, Bleeding History or Predisposition, Labile INR, Elderly, Drugs/Alcohol Concomitantly), and GARFIELD‐AF^13^, ^14^, ^15^ scores were calculated retrospectively.

All tests were two‐sided, with *p* < .05 considered as statistically significant. All analyses were carried out using R statistical software and SAS (version 9.4).

### 2.3 External validation

External validation was carried out with data from the Danish National Patient Register.^16^ This cohort comprised 39 929 patients enrolled nationwide between 2010 and 2015, within 10 days after a first diagnosis of AF, treated with OAC, and followed up for at least 2 years thereafter. Exclusion criteria were immigrant status, mortality within 10 days after discharge, or a history of rheumatic valve disease or previous valve interventions. ICH was defined as ICD‐10 codes I60, I61, and I62.^17^

We evaluated the discriminative performance of the GARFIELD/ORBIT‐AF ICH predictive model (henceforth known as the ICH predictive model) using Cox regression. Follow‐up was truncated at 2 years and a *C*‐statistic with 95% confidence interval (CI) was calculated using the coefficients obtained in the GARFIELD‐AF population. Calibration was assessed by calculating deciles of predicted probabilities and plotting the average predicted 2‐year rate versus the observed Kaplan–Meier rate and 95% CI within each decile. The R packages glm and ggplot were used for the analysis.

## 3 RESULTS

The study population comprised 53 261 anticoagulated patients: 34 306 in GARFIELD‐AF and 18 995 in ORBIT‐AF. ICH occurred in 284 patients: 158 in GARFIELD‐AF and 126 in ORBIT‐AF (Supporting Information: Figure S1). Baseline characteristics by ICH occurrence over 2 years are shown in Table 2. Data for individual registries are provided in Supporting Information: Figures S2 and S3 and Table S2.

**Table 2:** Baseline characteristics of study population by ICH occurrence over 2 years.

Variable	No ICH (*n* = 52 977)	Any ICH (*n* = 284)	*p* Value^a^
Sex, *n* (%)			.1752
Male	29 913 (56.5)	149 (52.5)	
Female	23 064 (43.5)	135 (47.5)	
Age, median (IQR) (years)	72.0 (64.0; 79.0)	77.0 (71.5; 83.0)	<.0001
Age group, *n* (%) (years)			<.0001
<65	13 316 (25.1)	31 (10.9)	
65–69	8440 (15.9)	29 (10.2)	
70–74	9469 (17.9)	44 (15.5)	
>75	21 752 (41.1)	180 (63.4)	
Ethnicity, *n* (%)			.3371
Caucasian	39 036 (74.9)	220 (78.6)	
Hispanic/Latino	3090 (5.9)	18 (6.4)	
Asian	8151 (15.6)	33 (11.8)	
Afro‐Caribbean/mixed/other	1870 (3.6)	9 (3.2)	
BMI, median (IQR) (kg/m^2^)	28.1 (24.7; 32.5)	27.1 (23.9; 31.3)	.0190
SBP, median (IQR) (mmHg)	130.0 (120.0; 142.0)	133.0 (120.0; 142.0)	.0809
DBP, median (IQR) (mmHg)	79.0 (70.0; 85.0)	78.0 (68.0; 84.0)	.1405
Pulse rate, median (IQR) (bpm)	80.0 (68.0; 96.0)	77.0 (68.0; 90.0)	.0798
Type of AF, *n* (%)			.4273
Permanent	7807 (14.7)	50 (17.6)	
Persistent	8744 (16.5)	40 (14.1)	
Paroxysmal	16 329 (30.8)	90 (31.7)	
New onset (unclassified)	20 095 (37.9)	104 (36.6)	
Cardiology unit diagnosis, *n* (%)	37 810 (71.4)	185 (65.1)	.0206
Medical history, *n* (%)			
Congestive heart failure	13 511 (25.5)	82 (28.9)	.1940
Coronary artery disease	12 504 (23.6)	88 (31.0)	.0035
Acute coronary syndromes	5745 (10.9)	43 (15.2)	.0199
Coronary artery bypass graft	3336 (6.3)	35 (12.3)	<.0001
Stenting	5246 (9.9)	41 (14.5)	.0107
Vascular disease^b^	14 496 (27.5)	107 (37.9)	<.0001
Prior stroke	4179 (7.9)	42 (14.8)	<.0001
Prior TIA	2977 (5.6)	33 (11.7)	<.0001
Prior bleeding^c^	1676 (3.2)	18 (6.4)	.0023
Hypertension	42 293 (79.9)	238 (84.1)	.0818
Hypercholesterolemia^d^	27 533 (52.9)	167 (60.1)	.0171
Diabetes	13 332 (25.2)	68 (23.9)	.6359
Cirrhosis^e^	542 (1.0)	2 (0.7)	.5884
Moderate‐to‐severe CKD^f^	9242 (19.2)	81 (30.7)	<.0001
Dementia^g^	835 (1.6)	6 (2.1)	.4738
Hyperthyroidism	922 (1.8)	2 (0.7)	.1764
Hypothyroidism	5472 (10.5)	35 (12.3)	.3036
Heavy alcohol use, *n* (%)	1303 (2.7)	4 (1.6)	.2447
Current smoker, *n* (%)	4376 (8.8)	19 (7.0)	0.3103
OAC at baseline, *n* (%)			<0.0001
NOAC	24 246 (45.8)	87 (30.6)	
VKA	28 731 (54.2)	197 (69.4)	
Concomitant AP, *n* (%)	13 352 (25.2)	98 (34.5)	.0003
CHA_2_DS_2_‐VASc, median (IQR)	3.0 (2.0; 4.0)	4.0 (3.0; 5.0)	<.0001
HAS‐BLED, median (IQR)^h^	1.0 (1.0; 2.0)	2.0 (1.0; 3.0)	<.0001

Abbreviations: AF, atrial fibrillation; BMI, body mass index; CHA_2_DS_2_‐VASc, congestive heart failure, hypertension, age ≥75 (doubled), diabetes, stroke (doubled), vascular disease, age 65–74 and sex category (female); CKD, chronic kidney disease; DBP, diastolic blood pressure; GARFIELD‐AF, Global Anticoagulant Registry in the FIELD‐Atrial Fibrillation; GI, gastrointestinal; HAS‐BLED, Hypertension, Abnormal Renal/Liver Function, Stroke, Bleeding History or Predisposition, Labile INR, Elderly, Drugs/Alcohol Concomitantly; ICH, intracranial hemorrhage; IQR, interquartile range; NOAC, nonvitamin K oral anticoagulant; ORBIT‐AF, Outcomes Registry for Better Informed Treatment of Atrial Fibrillation; SBP, systolic blood pressure; TIA, transient ischemic attack; VKA, vitamin K antagonist.

^a^ Calculated using *χ* ^2^ or Fisher exact test for categorical variables, as appropriate, and using Wilcoxon rank‐sum test for continuous variables.

^b^ Defined as peripheral artery disease and/or coronary artery disease.

^c^ Prior bleeding for GARFIELD‐AF, prior GI bleeding for ORBIT‐AF.

^d^ Hypercholesterolemia for GARFIELD‐AF, and hyperlipidemia for ORBIT‐AF.

^e^ Cirrhosis for GARFIELD‐AF, and liver disease for ORBIT‐AF.

^f^ Classified according to US National Kidney Foundation criteria.

^g^ Dementia for GARFIELD‐AF, and cognitive impairment/dementia for ORBIT‐AF.

^h^ Risk factor “labile INR” not included because not collected at baseline; hence, maximum HAS‐BLED score was 8 points, not 9.

ICH and non‐ICH patients differed in several baseline characteristics including median age, coronary artery disease, vascular disease, prior stroke/TIA, and moderate‐to‐severe chronic kidney disease (CKD) (estimated glomerular filtration rate [eGFR] less than 60 mL/min/1.73 m^2^; US National Kidney Foundation stages 3–5). The ICH event rate calculated over 2 years was 0.31 (95% CI: 0.28–0.35) per 100 person‐years (Supporting Information: Table S3). For GARFIELD‐AF only, sites of intracranial bleed (Supporting Information: Table S4) and distribution of ICH over 2 years (Supporting Information: Table S5) were also collected.

**Figure 1:**
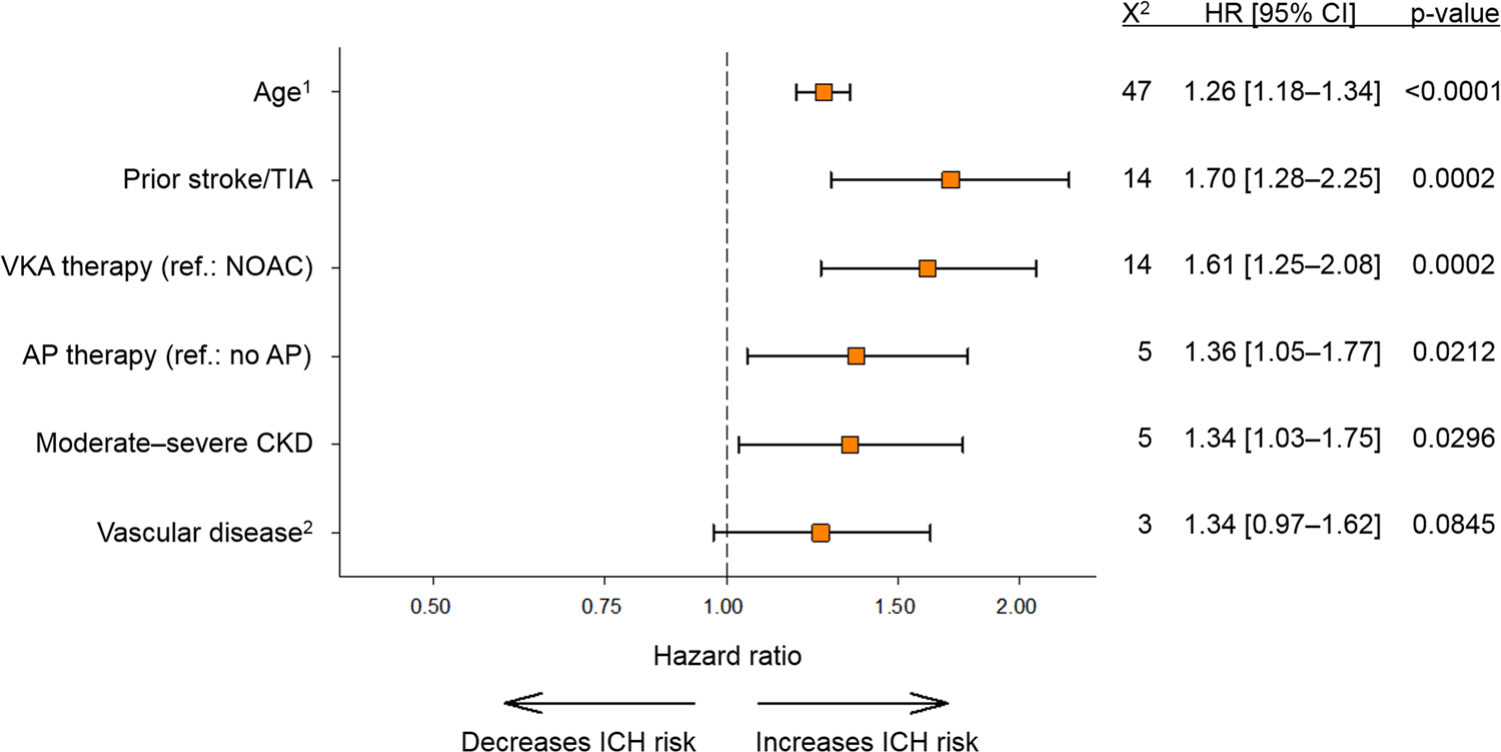
Hazard ratios (HRs), *χ* ^2^, and p values for components of the ICH predictive model. ^1^HR and 95% confidence interval (CI) were based on incremental units of “per 5‐year increase.” ^2^Vascular disease—peripheral artery disease and/or coronary artery disease. AP, antiplatelet treatment (acetylsalicylic acid and/or adenosine diphosphate inhibitors). CKD, chronic kidney disease; NOAC, nonvitamin K antagonist oral anticoagulant treatment; TIA, prior stroke/transient ischemic attack; VKA, vitamin K antagonist.

Independent predictors of ICH were age, prior stroke/TIA, VKA versus NOAC therapy, concomitant antiplatelet (AP) use, and moderate‐to‐severe CKD (Figure 1). VKA therapy was associated with 61% higher risk of ICH compared with NOAC: 0.38 (95% CI: 0.33–0.43) versus 0.22 (95% CI: 0.18–0.27) per 100 person‐years, respectively (*p* < .0001). Rates of ICD were also higher in patients with CKD, in particular for moderate‐to‐severe disease, although these trends did not reach statistical significance (Supporting Information: Table S6). In addition, ICH events per 100 person‐years occurred at a significantly higher rate in patients on OAC plus AP (0.43; 95% CI: 0.36–0.53) than in patients treated with OAC alone (0.27; 95% CI: 0.23–0.31; *p* = .0001). Although vascular disease was not a significant independent predictor of ICH multivariate analysis (*p* = .085), it was selected by LASSO methodology and improved the performance of the ICH predictive model. All components were also predictive of major bleeding (Supporting Information: Table S7). When comparing ICH predictors in the two patient registries, ORBIT‐AF patients were more frequently treated with AP and had higher rates of moderate‐to‐severe CKD and vascular disease. Conversely, VKA treatment was more common in GARFIELD‐AF (Supporting Information: Table S8).

**Figure 2:**
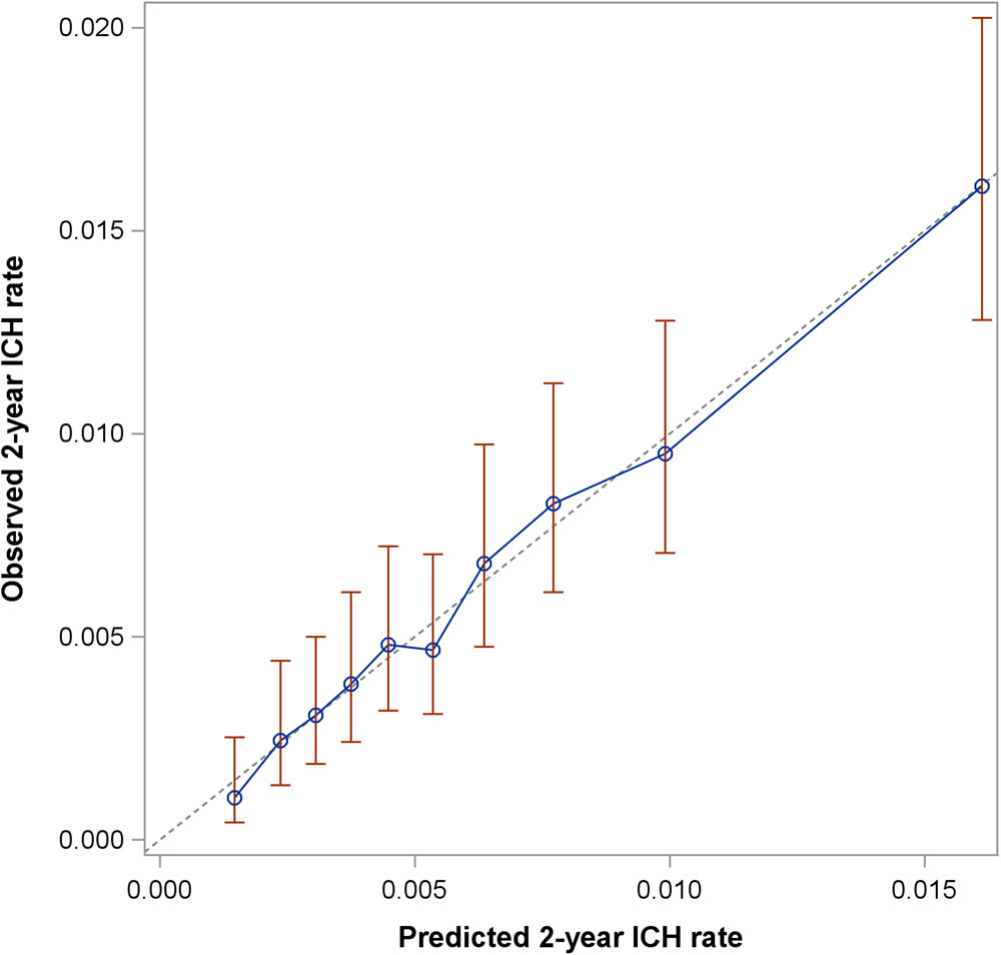
Calibration of the intracranial hemorrhage (ICH) predictive model at 2 years follow‐up. Predicted versus observed 2‐year ICH probabilities in the training data set.

The ability of the model to discriminate individuals with and without ICH was fair, with optimization corrected *C*‐statistics 0.68 (95% CI: 0.65–0.71) at 2 years (Table 3), 0.67 (95% CI: 0.63–0.71) at 1 year, and 0.71 (95% CI: 0.61–0.81) at 30 days. Calibration between predicted and observed ICH probabilities was good (Figure 2).

**Table 3:** Comparison of the ICH predictive model with previously published models and their components.

Population	*N*	ICH predictive model^a^	CHA_2_DS_2_‐VASc score	HAS‐BLED score	GARFIELD‐AF bleeding score^b^
GARFIELD‐AF	34 306	0.67 (0.63–0.71)	0.60 (0.56–0.64)	0.60 (0.56–0.64)	0.63 (0.59–0.67)
ORBIT‐AF	18 955	0.70 (0.65–0.75)	0.66 (0.61–0.71)	0.63 (0.58–0.67)	0.66 (0.61–0.71)
Total	53 261	0.68 (0.65–0.71)	0.63 (0.60–0.66)	0.62 (0.59–0.65)	0.65 (0.62–0.68)

*Note*: *C*‐statistics (95% CI) for discriminating patients with or without ICH.

Abbreviations: AF, atrial fibrillation; AP, antiplatelet treatment; CI, confidence interval; CHA_2_DS_2_‐VASc, congestive heart failure, hypertension, age ≥75 (doubled), diabetes, stroke (doubled), vascular disease, age 65–74 and sex category (female); CKD, chronic kidney disease; GARFIELD‐AF, Global Anticoagulant Registry in the FIELD‐Atrial Fibrillation; HAS‐BLED, Hypertension, Abnormal Renal/Liver Function, Stroke, Bleeding History or Predisposition, Labile INR, Elderly, Drugs/Alcohol Concomitantly; ICH, intracranial hemorrhage; OAC, oral anticoagulant.

^a^ Optimism corrected by subtracting the estimate of optimism in 100 bootstrap samples from the original estimate of the *C*‐statistic.

^b^ Based on the GARFIELD risk score for prediction of major bleeding/hemorrhagic stroke; score includes age, OAC treatment, moderate‐to‐severe CKD, history of bleeding, pulse, AP treatment, diabetes, vascular disease, and carotid occlusive disease.

The ICH predictive model compared favorably with CHA_2_DS_2_‐VASc, HAS‐BLED, and GARFIELD‐AF scores for discriminating patients with incident ICH on follow‐up (Table 3).

The 1‐ and 2‐year outcomes can be calculated by applying the formulas in Supporting Information: Table S9. For 2‐year outcomes, a simple scoring table and nomogram are provided.

The model was validated in an independent cohort of 39 929 AF patients from the Danish National Patient Register, 238 of which experienced an ICH within 2 years of AF diagnosis (baseline characteristics; Supporting Information: Table S10). The *C*‐statistic in this cohort was 0.65 (95% CI: 0.62–0.68). Calibration was reasonable, and best for cases with lower moderate risk (Supporting Information: Figure S4).

## 4 DISCUSSION

Our study of more than 53 000 prospectively observed AF patients identified major risk factors for ICH, the most serious complication of anticoagulation. We developed a model which successfully predicted the probability of ICH in a combined cohort of GARFIELD‐AF and ORBIT‐AF patients, as well as in an independent validation data set. This model may therefore aid risk stratification and shared decision‐making among clinicians and patients.

### 4.1 Comparison to previous studies

Real‐world data and pivotal clinical trials of OACs point to similar risk factors for ICH: age, prior stroke/TIA, concomitant AP use, and VKA versus NOAC therapy.^6^, ^7^, ^18^, ^19^ The ROCKET (Rivaroxaban Once‐daily oral direct factor Xa inhibition Compared with vitamin K antagonism for prevention of stroke and Embolism Trial) trial also identified reduced serum albumin and platelet count,^6^ two parameters that were not available for patients in this study. Moreover, an increased ICH risk associated with Asian or Black ethnicity was reported in clinical trials,^5^, ^20^, ^21^, ^22^ as well as in a retrospective study of patients treated with warfarin.^23^ By contrast, race was not a risk factor in the pooled GARFIELD‐AF/ORBIT data set. Indeed, ICH occurred even slightly less frequently in Asian patients compared to patients from other ethnic groups. A possible explanation for this discrepancy lies in different frequencies of prior stroke: in the ROCKET trial,^20^, ^21^ this major risk factor for ICH was more prevalent in Asian (65%) versus non‐Asian patients (54%), whereas the ratio within the combined GARFIELD‐AF/ORBIT cohort was 10.0% Asians versus 7.6% non‐Asians. Thus, the randomized controlled trial (RCT) cohorts had higher baseline risk for ICH, which might accentuate differences in risk. Furthermore, Asians in GARFIELD‐AF/ORBIT often received lower NOAC doses, possibly reducing the rate of adverse bleeding events relative to other ethnic groups.^1^, ^24^

Rates of ICH were lower in GARFIELD‐AF compared to ORBIT‐AF. We consider under‐reporting an unlikely cause because data in GARFIELD‐AF were thoroughly audited and quality controlled.^25^ Rather, the reason for this discrepancy might be differences in the baseline characteristics: apart from VKA use, all ICH predictors in our model were more prevalent in ORBIT‐AF than GARFIELD‐AF patients.

In addition, the ICH event rate of the pooled GARFIELD‐AF/ORBIT‐AF population was considerably lower than the rates observed in RCTs of NOACs,^26^, ^27^ This too might reflect higher baseline risks because clinical trial cohorts are typically designed to recruit patients at higher risk of adverse events. A lower incidence of ICH was also seen in other registry studies investigating bleeding in AF patients. For example, just 15 ICH events occurred among 7243 patients in the European Registry in Atrial Fibrillation (PREFER in AF) over 1 year.^28^ Therefore, risk factors specifically related to ICH were not evaluated. By combining two large registries, we were able to observe 284 ICH events, an adequate number for studying predictors and modeling risk in a real‐world population. Although developing a risk score for major bleeding in AF patients^29^, ^30^ was not the aim of this study, we found that most ICH predictors were shared by both conditions, which consequently might be treatable using similar strategies.

### 4.2 CKD and VKA use

Prior trials which excluded cases with severe CKD showed lower rates of major bleeding in patients treated with NOACs compared to warfarin regardless of CKD severity,^31^ and lower rates of ICH in patients with mild‐to‐moderate CKD.^32^ The present analysis identified moderate‐to‐severe CKD, characterized by an eGFR < 60 mL/min/1.73 m^2^, as an independent risk factor for ICH in AF patients on OAC therapy. This is consistent with previous reports highlighting an eGFR < 30 mL/min/1.73 m^2^ as a major predictor for general bleeding risk in patients treated with warfarin^33^, ^34^ or NOAC,^35^ but recommending NOACs in patients with an eGFR > 60 mL/min/1.73 m^2^.

The present analysis extends these findings, showing that VKA was associated with a 61% higher risk of ICH compared to NOACs in a cohort that included patients with advanced and severe CKD. The difference between NOAC and VKA was similar in the subgroup with moderate‐to‐severe CKD, and smaller in patients with mild or no CKD. However, these trends did not reach statistical significance, likely because few events were observed. Together with the finding that VKA use (reference: NOACs) and a higher severity of CKD were independent predictors of ICH, it suggests that NOACs are associated with fewer ICHs than VKA in patients with moderate‐to‐severe CKD. This might be pertinent because these patients were excluded from landmark NOAC trials and therefore generally receive VKAs instead of NOACs, potentially exposing them to an increased risk. However, because this study was designed to predict ICH risk and not for detecting causal relationships, confirmation will be needed.

### 4.3 Dual therapy increased ICH risk

OAC and aspirin are still widely given together, often due to coexisting coronary artery disease, despite evidence that it increases the risk of bleeding without reducing the risk of stroke or systemic embolism, including in AF patients with prior stroke/TIA.^36^, ^37^, ^38^ Concomitant OAC and AP therapy was also a predictor of ICH risk in RE‐LY (Randomized Evaluation of Long‐Term Anticoagulation Therapy trial),^7^ ARISTOTLE (Apixaban for Reduction in Stroke and Other Thromboembolic Events in Atrial Fibrillation),^26^ and this study, further supporting that combined use of these drugs in AF patients should be avoided, if possible.

### 4.4 Risk prediction for ICH

Despite efforts to develop better models,^39^ an unmet need exists to reliably predict the risk of ICH in AF patients on OAC. Importantly, the model predicted ICH at least as well as the widely used HAS‐BLED and GARFIELD‐AF bleeding scores, as well as the CHA_2_DS_2_‐VASc score, which was primarily designed for determining stroke risk, but is familiar to practitioners and has repeatedly demonstrated the ability to predict the risk of OAC‐related bleeding events such as ICH.^40^ The discrimination of our model (*C*‐statistics 0.68; 95% CI: 0.65–0.71) also compared well with the performance of published models assessed by a recent NICE (National Institute for Health and Care Excellence) report,^41^ and an ICH prediction model that included clinical and genetic factors (*c*‐index: 0.58; 95% CI: 0.52–0.64 for events at 10 years).^39^ The minimal change in the *C*‐statistic after optimism adjustment indicates that the model might work well in other similar populations. Because the ICH score relies on data generally found in electronic medical records, it offers the potential for automatically alerting the treating clinician to high‐risk patients in need of closer monitoring.

### 4.5 Strengths and limitations

Pooling data from two large international prospective registries with an adequate proportion of ICH events allowed a meaningful assessment of risk factors. External validation in a nationwide Danish cohort supported the validity and generalizability of the ICH predictive model to other populations.

A limitation of this study was that data for some key factors of other predictive models were not available for all patients, namely, ethnicity, ICH subtypes, or time in the therapeutic range for warfarin. Moreover, the ORBIT bleeding risk score was not available for patients enrolled in the GARFIELD‐AF registry as information on hemoglobin, hematocrit, or anemia was not collected.

## 5 CONCLUSION

This study identified factors associated with the risk of ICH in patients with AF treated with OACs in everyday clinical practice. Its results support selecting NOAC rather than VKA, and avoiding unnecessary use of concomitant AP therapy for reducing ICH risk in accordance with the latest ACC/AHA/HRS guideline recommendations. Moderate‐to‐severe CKD was confirmed as an independent predictor for ICH, demonstrating that anticoagulation in patients with this comorbidity requires careful consideration.

A new predictive model was developed for improving ICH risk stratification and shared decision‐making among clinicians and AF patients regarding OAC therapy. It relies on six clinical and demographic predictors requiring no specialized tests. The model performed well in internal validation, showed reasonable discrimination and calibration in an independent validation cohort, and was compared favorably with other scores whose use for ICH prediction was reported.

## CONFLICTS OF INTEREST STATEMENT

Toon Wei Lim has received research support from Bayer, Boehringer Ingelheim, and Pfizer. Alan John Camm has received institutional grants and personal fees from Bayer, Boehringer Ingelheim, Pfizer/BMS, and Daiichi Sankyo and personal fees from Portola. Daniel E. Singer has received research grants from Boehringer Ingelheim and Bristol‐Myers Squibb and consulting fees from Boehringer Ingelheim, Bristol‐Myers Squibb, Johnson & Johnson, Merck, and Pfizer. Jean‐Pierre Bassand reports personal fees from Thrombosis Research Institute. Gregg C. Fonarow has consulted for Abbott, Bayer, Janssen, Medtronic, and Novartis. Keith A.A. Fox has received grants and personal fees from Sanofi, Bayer, and Anthos. Michael Ezekowitz has consulted for Boehringer Ingelheim, Daiichi Sankyo, Bristol‐Myers Squibb, and Janssen Scientific Affairs. Bernard J. Gersh is a member of Data Safety Monitoring Board–Mount Sinai St. Luke's, Boston Scientific Corporation, Teva Pharmaceutical Industries, St. Jude Medical, Janssen Research & Development, TRI, Duke Clinical Research Institute, Duke University, Kowa Research Institute, Cardiovascular Research Foundation, and Medtronic and consults for Janssen Scientific Affairs, Xenon Pharmaceuticals, and Sirtex Medical. Elaine M. Hylek consults for Bayer, Boehringer‐Ingelheim, Bristol‐Myers‐Squibb, Janssen, Medtronic, and Pfizer. Ajay K. Kakkar has received research support from Bayer AG and Sanofi; personal fees from Bayer AG, Janssen Pharma, Pfizer, Sanofi, Verseon, and Anthos Therapeutics. Kenneth W. Mahaffey has received research support from Afferent, Amgen, Apple, AstraZeneca, Cardiva Medical, Daiichi Sankyo, Ferring, Google (Verily), Johnson & Johnson, Luitpold, Medtronic, Merck, NIH, Novartis, Sanofi, St. Jude, and Tenax, and reports consulting or other services for Abbott, Ablynx, AstraZeneca, Baim Institute, Boehringer Ingelheim, Bristol‐Myers Squibb, Elsevier, GSK, Johnson & Johnson, Medergy, Medscape, Mitsubishi, Myokardia, NIH, Novo Nordisk, Portola, Radiometer, Regeneron, SmartMedics, Springer, and UCSF. Karen S. Pieper has consultancies with Johnson & Johnson, Element Science, Artivion, and Novartis. Eric D. Peterson has received research grants from Janssen Pharmaceuticals and Eli Lilly and consulted for Janssen Pharmaceuticals and Boehringer Ingelheim. Jonathan P. Piccini has received grants for clinical research from Abbott, American Heart Association, Bayer, Boston Scientific, Janssen Pharmaceuticals, NHLBI, and Philips and serves as a consultant to Abbott, Allergan, ARCA Biopharma, Biotronik, Boston Scientific, Johnson & Johnson, LivaNova, Medtronic, Milestone, Sanofi, Philips, and Up‐to‐Date. The remaining authors declare no conflict of interest.

## Supporting information

Supporting information.

## Data Availability

Requests for patient‐level data can be made to the head of statistics at the Thrombosis Research Institute (svirdone@tri-london.ac.uk). These requests should include a protocol summary and a summary of the statistical analysis plan. The request will be reviewed by the data‐sharing committee for approval and the next steps will be discussed with the requestor.
